# Juvenile Fibrosarcoma of the Parotid Gland in an Adult Patient: A Case Report

**DOI:** 10.7759/cureus.105994

**Published:** 2026-03-27

**Authors:** Mariana Escobar Howard, Diana Carolina Moreno Jiménez, Álvaro Diego García Valencia

**Affiliations:** 1 Otolaryngology - Head and Neck Surgery, Universidad de Antioquia, Medellín, COL; 2 Head and Neck Surgery, Hospital Pablo Tobón Uribe, Medellín, COL

**Keywords:** juvenile fibrosarcoma, parotidectomy, parotid gland, salivary gland, sarcoma

## Abstract

Salivary gland sarcomas are rare neoplasms, with the parotid gland being the most frequent site of involvement. Among these, fibrosarcoma represents an exceptional variant. Its diagnosis is primarily through histopathological and immunohistochemical analysis. Its presentation in the parotid gland is rare, and the occurrence of the infantile-juvenile subtype in an adult patient has not been previously described.

In this clinical setting, we report the case of a 29-year-old man presenting with a rapidly growing right preauricular mass and ipsilateral facial paresis. Computed tomography revealed a solid lesion involving the superficial and deep lobes of the parotid gland, extending into adjacent spaces with facial nerve involvement, without distant metastasis. Total parotidectomy with selective neck dissection was performed. Histopathological examination demonstrated a spindle-cell neoplasm with perineural invasion. Immunohistochemistry was negative for epithelial, neural, and muscular markers, with focal smooth muscle actin positivity and a low proliferative index (Ki-67: 4%), consistent with juvenile fibrosarcoma.

The case was evaluated by a multidisciplinary team, and after complete surgical resection with negative margins, adjuvant radiotherapy was recommended due to the high risk of local recurrence.

## Introduction

Primary sarcomas of the salivary glands are rare neoplasms, accounting for 0.3% to 1.5% of all tumors in these glands, with the parotid gland being the most frequent site of presentation [1]. Within this group, fibrosarcoma is an uncommon entity, defined by the World Health Organization (WHO) as a malignant tumor characterized by interlacing fascicles of collagen fibers in the absence of other histological structures, such as bone or cartilage [2]. It is composed of fibroblasts that exhibit no other lines of differentiation, thus rendering it a diagnosis of exclusion [3]. Consequently, its diagnosis relies on histopathological examination, supplemented by immunohistochemistry and molecular testing, to differentiate it from other spindle cell tumors [4]. These primarily include monophasic synovial sarcoma, myofibroblastic sarcoma, malignant peripheral nerve sheath tumor (MPNST), high-grade myxofibrosarcoma, and leiomyosarcoma, among others [3].

The limited number of published cases and their clinical similarity to more frequent tumors in this region make preoperative diagnosis difficult, and well-established therapeutic guidelines are lacking. We present the case of an adult patient with juvenile fibrosarcoma of the parotid gland, an unusual presentation regarding both location and patient age, aiming to provide clinical, surgical, and histopathological information to aid in the recognition and management of this rare entity.

## Case presentation

A 29-year-old male patient, with a history of smoking and daily marijuana use and no other relevant personal history, consulted the Head and Neck Surgery service for a four-month clinical course characterized by the appearance of a rapidly growing large mass in the right preauricular region.

Physical examination revealed weakness of the right facial musculature, corresponding to a Grade II/VI facial paralysis according to the House-Brackmann scale [5]. Upon palpation, a stony-hard mass dependent on the right parotid gland was identified, measuring approximately 5 × 6 cm and adhering to deep planes.

The patient had previously consulted a general surgeon, where a neck ultrasound was requested; the initial report suggested a pleomorphic adenoma of the right parotid. Fine-needle aspiration (FNA) was also performed, describing stromal proliferation with areas of hyalinosis, which did not allow for a clear determination of the lesion's nature.

**Figure 1:**
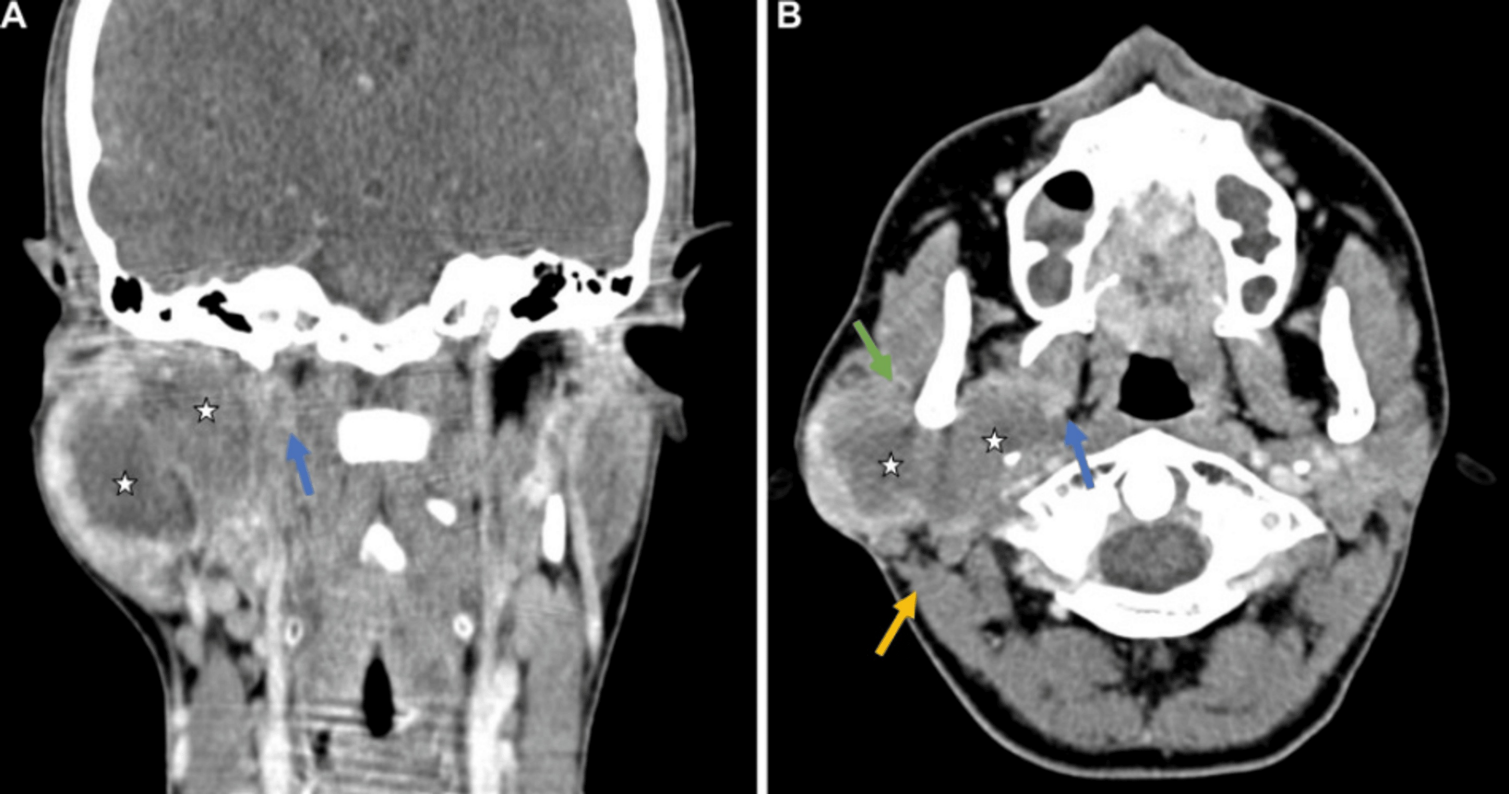
Contrast-enhanced computed tomography of the neck (A) Coronal view. (B) Axial view. A 5.6 cm solid, irregular lesion in the superficial and deep lobes of the right parotid gland, demonstrating peripheral enhancement and central areas of necrosis (stars). Infiltration of the parapharyngeal space and the medial pterygoid muscle is observed (blue arrows). The lesion contacts the posterior margins of the masseter (green arrow) and sternocleidomastoid (yellow arrow) muscles.

Further studies were supplemented with plain and contrast-enhanced computed tomography (CT) of the neck and chest. These reported a solid, irregular 5.6 cm lesion in the superficial and deep lobes of the right parotid gland, with infiltration of the parapharyngeal space and medial pterygoid muscle. Additionally, contact was noted with the posterior margin of the masseter, sternocleidomastoid, and the posterior belly of the digastric muscle; due to its extent, high involvement of the facial nerve was observed. No lymphadenopathy or signs of metastatic involvement were evident in the chest (Figure 1).

Given the rapid evolution of the lesion, clinical characteristics, and imaging findings highly suggestive of malignancy, the decision was made to proceed with surgical management for diagnostic and therapeutic purposes, performing a total parotidectomy associated with selective neck dissection.

During the procedure, a locally advanced tumor of approximately 5.6 cm was evidenced, lacking cleavage planes with adjacent structures. It involved the superficial and deep lobes of the parotid gland, with infiltration of the parapharyngeal space and the medial pterygoid, masseter, sternocleidomastoid, and posterior belly of the digastric muscles. The facial nerve was completely infiltrated by the tumor mass.

The histopathological study reported a spindle cell neoplasm with storiform and "herringbone" patterns, with neural involvement present. Resection margins were free of lesion; vascular invasion was absent, and periparotid and level IIA, IIB, and III lymph nodes showed no evidence of tumor involvement.

**Figure 2:**
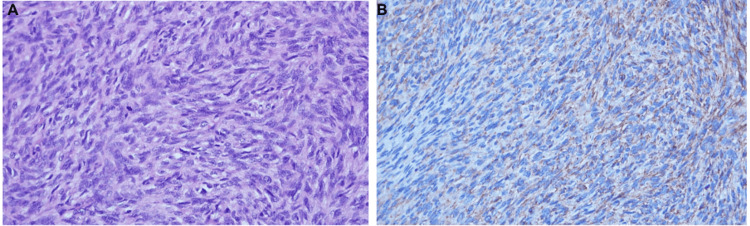
Histopathological study (A) Neoplastic cells showing a spindle cell pattern (H&E, ×40). (B) Neoplastic cells with focal positivity for beta-catenin (IHC, ×40).

In the immunohistochemical study, markers SSX/SS18, TLE1, CK, BCL2, CD99, S-100 protein, p63, STAT6, H-caldesmon, and desmin were negative. The Ki-67 proliferation index was 4%. Focal positivity for smooth muscle actin (SMA) and focal, very weak positivity for β-catenin were observed. These findings, in conjunction with the morphological characteristics, were compatible with juvenile fibrosarcoma (Figure 2).

The postoperative period was uneventful. The case was discussed in a tumor board with oncology and radiotherapy services. They indicated that the patient would benefit from adjuvant external beam radiation therapy using the intensity-modulated radiation therapy (IMRT) technique on the tumor bed to deliver a dose of 60 Gy in 30 fractions, as it is considered a high-risk tumor for local recurrence due to its classification as a soft tissue sarcoma, its size greater than 5 cm with perineural involvement, and its location in the head and neck.

## Discussion

Fibrosarcomas are malignant neoplasms originating from mesenchymal cells [6]. Depending on the age of presentation, they can be infantile, including juvenile and congenital, the latter identified in up to 40% of patients [6], or adult-type, the latter typically having a worse prognosis due to a higher risk of recurrence, metastatic incidence, and lower overall survival [7]. Infantile fibrosarcomas are characterized by rapidly progressive, painless growth that compresses adjacent structures, predominantly occurring in males [6] and in the extremities. In the head and neck, they are rare, ranging from 0.05% to 10% in some reviews [8,9], and their presentation in salivary glands is even more infrequent [10].

Although uncommon, adult-type presentation has been described in children at the abdominopelvic level [6]; however, infantile/juvenile presentation in adults is even rarer, and its frequency decreases further when located in the head and neck. Imaging, such as CT and MRI, can help determine the extent; histologically, both adult and infantile fibrosarcomas share some characteristics. However, infantile fibrosarcoma exhibits less pleomorphism, uniform spindle cells, minimal atypia despite frequent mitosis, and infiltrative growth with lower aggressiveness [11]. Immunohistochemistry for infantile fibrosarcoma may show positivity for vimentin and SMA, the latter of which was also evident in this case, as well as a low Ki-67 index [11-14]. S-100 and desmin are usually negative, as observed in our case; CD34 was not assessed in this patient, although the literature describes occasional focal positivity [9,15]. Adult fibrosarcoma, in contrast, has been associated with a higher Ki-67 index [14], negative SMA, desmin, S100, and CD34, greater cellular atypia [16], and vimentin positivity (Table 1) [17].

**Table 1:** Immunohistochemical markers in juvenile vs. adult-type fibrosarcoma It should be noted that adult-type fibrosarcoma is an immunohistochemical diagnosis of exclusion, established only when all markers for other soft tissue tumors yield negative results.

Histology: Spindle cells with elongated nuclei [9]		
Immunohistochemical marker	Juvenile/infantile	Adult-type
Vimentin	Positive [12]	Positive [17]
Smooth muscle actin (SMA)	May be positive or negative [11-13]	Negative [16]
Desmin	May be positive or negative [11-13]	Negative [16]
S100	May be positive or negative [12,13]	Negative [16]
CD34	May be positive or negative [7,15]	Negative [16]
Ki-67	Low to Moderate [14]	High [14]

The definitive diagnosis may be supported by molecular testing, particularly the identification of NTRK rearrangements such as ETV6-NTRK3 fusion [13]; this could not be completed in our case due to administrative constraints. However, the evaluated immunohistochemistry allowed for an oriented diagnosis to establish complementary treatment and define follow-up.

The differential diagnosis of fibrosarcoma encompasses various soft tissue tumors exhibiting similar spindle cell histology [18]. Regarding infantile fibrosarcoma, the primary differentials include infantile myofibroma, composite fibromatosis, dermatofibrosarcoma protuberans, spindle cell rhabdomyosarcoma, infantile rhabdomyofibrosarcoma, and primitive myxoid mesenchymal tumor of infancy [19]. Conversely, the differential diagnosis for adult-type fibrosarcoma predominantly involves monophasic synovial sarcoma, solitary fibrous tumor, myofibroblastic sarcoma, MPNST, high-grade myxofibrosarcoma, leiomyosarcoma, fibrosarcomatous dermatofibrosarcoma protuberans, low-grade fibromyxoid sarcoma, and undifferentiated pleomorphic sarcoma, among others [3]

Treatment is based on complete surgical excision; once negative margins are obtained, only close follow-up is required. In large tumors, adjuvant chemotherapy with vincristine or actinomycin has been described [7] and may also be considered postoperatively in cases of close or positive margins, or when complete surgical excision is not feasible because of tumor extent [8,12,16,17]. Radiotherapy is not typically required, although it may be considered in selected cases within the broader context of soft tissue sarcomas [20]. Reported overall survival reaches up to 93% at five years, with recurrence rates of up to 43% and metastasis in up to 8%, predominantly involving the lungs and lymph nodes [7].

Parotid sarcomas are extremely rare malignant entities. Survival depends on treatment, which may include surgical resection, radiotherapy, chemotherapy, or a combination thereof. Surgery is the only modality consistently associated with improved survival, and management depends on the extent of local and distant disease [1]. To date, there are no reports in the literature of juvenile fibrosarcoma in the parotid gland; therefore, treatment is based on the available evidence, with the aim of minimizing morbidity, improving survival, and expanding the literature on this pathology.

## Conclusions

Juvenile fibrosarcoma of the parotid presenting in adulthood has not been reported in the literature to date. Its extremely rare presentation poses significant challenges in both diagnosis and management. Therefore, we emphasize the importance of a multidisciplinary approach to ensure timely and adequate diagnosis, avoiding therapeutic delays and thus improving patient survival.
